# EFFECT OF TRANEXAMIC ACID ON BLEEDING CONTROL IN TOTAL KNEE ARTHROPLASTY

**DOI:** 10.1590/1413-785220162403149200

**Published:** 2016

**Authors:** DAVID SADIGURSKY, DANIEL ANDION, PÉRICLES BOUREAU, MARIA CORDULINA FERREIRA, ROGÉRIO JAMIL FERNANDES CARNEIRO, PAULO OLIVEIRA COLAVOLPE

**Affiliations:** 1. Faculdade de Tecnologia e Ciências de Salvador (FTC), Salvador, BA, Brazil; 2. Centro de Estudos em Ortopedia e Traumatologia (CEOT-COT), Orthopedic and Traumatology Clinic, Salvador, BA, Brazil

**Keywords:** Tranexamic acid, Artrhoplasty, replacement, knee, Knee, Hemorrhage

## Abstract

**Objectives:**

To analyze the effectiveness of intravenous (IV) tranexamic acid (TA) in reducing blood loss in total knee arthroplasty (TKA).

**Method:**

The population sample was composed of patients with a diagnosis of primary knee osteoarthritis. The patients undergoing TKA were divided in two groups. Group A: comprised patients who used IV TA and B group, formed by patients who did not use TA in the intra or post-operative period. For descriptive analysis, quantitative variables were represented by mean and standard deviations when their distribution was normal and interquartile ranges and medians for non-normal variables.

**Results:**

The mean age of patients was 68 years old, most of them were female and with involvement of the left knee. Postoperatively patients who had used IV TA showed less bleeding rate and less hemoglobin rate reduction.

**Conclusion:**

The use of IV TA in TKA reduces blood loss in peri- and postoperative periods. Regarding total blood loss reduction, hemoglobin rate and need for blood transfusions, IV TA should be used routinely during TKA since it has been shown to be safe with no increase in side effects as thromboembolic events. ***Level of Evidence III. Retrospective Comparative Study.***

## INTRODUCTION

Total knee arthroplasty (TKA) is one of the most common orthopedic procedures for the treatment of advanced osteoarthritis. Since 2010, more than 600.000 TKA have been performed annually in the United States and are becoming increasingly common.^1^

TKA, as any surgical procedure, is prone to a number of postoperative complications, including considerable blood loss, reaching about 1000 to 1500 ml after a conventional knee arthroplasty,^2^ blood transfusion being necessary in some cases. Along with blood loss, higher rates of postoperative anemia, increased predisposition to cardiopulmonary events, and increased cost for prolonged hospitalization could be identified, as well as complications related to blood transfusion such as immune reactions and infection, besides decreased rate of patient satisfaction.^3^ ^-^ ^5^

Other possible complications after TKA identified in the literature include: infections of the operative site, peripheral venous thromboembolism and pulmonary embolism, vascular lesions, aseptic loosening, exacerbation of pre-existing clinical diseases and death due to clinical and surgical complications.^6^ ^,^ ^7^

In order to minimize a major complication of TKA, which is intra postoperative bleeding, some alternatives are constantly being studied. Among them, the use of intravenous or topic tranexamic acid (TA), epinephrine, fibrin glue, Floseal^(r)^ and autotransfusion.^8^ The analysis of the clinical efficacy of TA use in reducing blood loss in TKA is necessary, as the current literature shows no consensus on the more effective route of administration and dose. Recent studies show that TA is a key ally in reducing bleeding.^9^

TA, a synthetic antifibrinolytic agent, has in its formula the trans isomer of 4-amino-methyl-cyclohexane carboxylic, commercialized as Transamin^(r)^ in Brazil, which is the synthetic derivative from the amino acid lysine. This formula has a strong attraction to the lysine binding sites on plasminogen and plasmin, and by competitively inhibiting activation and action of plasmin.^8^ Its action is mainly based on slowing the fibrinolysis process (a potent inhibitor of fibrinolytic activity of plasmin), a subsequent phase of clot formation, when the dissolution time of the fibrin network is extended. The clot is, then, preserved and does not result in activation of the coagulation cascade. These properties enhance the hemostatic effectiveness of the substance by reducing the intensity and risk of bleeding in surgical procedures, trauma and bleeding prone disease. TA is rapidly absorbed. Approximately 90% of an IV dose is excreted in urine in 24h, it has a plasma half-life of approximately 2h, maintaining therapeutic levels for 6-8h. Its action preserves the hemostatic clot making hemostasis more efficient, reducing the intensity and risk of bleeding either IV or topically administered. This study is justified in view of the need to obtain a reduction of bleeding in TKA procedures, avoiding increased complications in the postoperative period. The aim, therefore, is to evaluate the introduction of TA in TKA surgery, to reduce blood loss and hemoglobin reduction, consequently decreasing in the length of hospitalization and transfusion requirements.

## MATERIALS AND METHODS

The research model adopted for this trial was an observational retrospective cohort study. The population sample was composed of 59 patients with a diagnosis of primary osteoarthritis of the knee, submitted to surgical treatment of TKA, between January 2008 and June 2014.

To participate in the study patients were of both genders were selected, aged 60-80 years, with Ahlback radiological classification between IV and V, who had knee pain above seven ​​assessed by visual analogue pain scale (VAS)^10^, and agreed to participate in the study by signing a Free and Informed Consent form, to be operated and inserted a primary prosthesis cemented knee, classified by the American Society of Anesthesiologists as groups 1 and 2.

The criteria used for sample analysis were the anthropometric data of patients, range of motion, operated side, comorbidities, previous history of stroke, myocardial infarction or deep vein thrombosis/lung thromboembolism. To assess the patients' functions, the WOMAC^11^ questionnaire (Western Ontario and McMaster Osteoarthritis Index) and VAS for pain were used.

Exclusion criteria were patients with history of coagulopathy, pulmonary embolism, users of anticoagulants prior to surgery or anti-inflammatory steroid up to 2 days before surgery, diagnosed with bone tumors, acute myocardial infarction, brain stroke, chronic arterial disease or hemoglobin levels below 10 g/dl, patients with contraindications to TA such as those with history of stroke, myocardial infarction or pulmonary embolism.

The patients were divided into two groups. Group A, which comprised patients who used 10mg/kg IV TA after cementation of the components, and had the dose repeated 3h after the first dose. Group B, formed by patients who did not use TA. Both groups used a tourniquet and the deep and superficial planes were sutured with no withdrawal until complete coverage of the wound with dressing. The suction drain was used in all patients and removed after 48h, suction being reviewed every 6h.

The parameters analyzed were: 1) Losses through the suction drain installed after TKA in 24 and 48h; 2) Hemoglobin levels before and after the surgical procedure at 6, 24 and 48h; 3) Need for blood transfusion at 24 and 48h postoperatively, performed when hemoglobin levels were below 8 g/dl; 4) Reduction of complications such as infection, hemolytic disorders, myocardial infarction, stroke, deep vein thrombosis/lung thromboembolism; 5) Early rehabilitation with analysis of range of motion and ability to ambulate; and 6) Reduction of hospitalization time.

For descriptive analysis, quantitative variables were represented by mean and standard deviation when their distributions were normal and interquartile range and median when they were not normal. Categorical variables were represented by frequencies and percentages.

Numerical variables were compared between groups using the Student *t* -test for normal distribution variables and Mann-Whitney test for non-normally distributed variables. Proportion comparisons were made using the Chi Square test or Fisher's exact test.

The analyzes were conducted by IBM Statistical Package for Social Sciences (SPSS, Chicago, IL, USA) version 20.0 and the language and environment programming R.

The study was submitted to the guidelines of Resolution 466/12 on research involving human subjects of the Brazilian National Health Council. The project was submitted to the Ethics Committee of *Faculdade de Tecnologia e Ciências* , Salvador, BA, Brazil and approved under n^o^ 897.001.

## RESULTS

**Figure 1:**
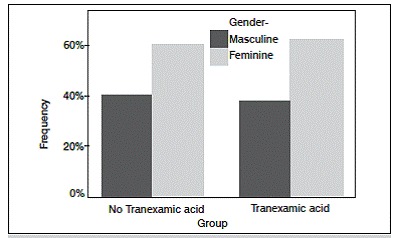
Gender frequency by group.

**Figure 2:**
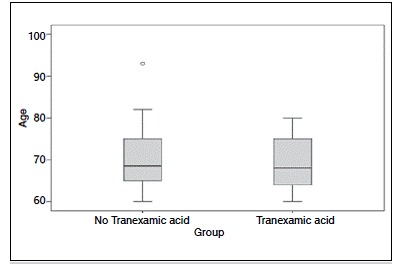
Age by group.

We evaluated 59 patients undergoing TKA performed by the same surgeon. Table 1 shows the general data of patients participating in the trial. Regarding gender there were 36 (61%) female participants and 23 (39%) males. (Figure 1) Regarding age, the median was 68.5 years old in group A and 68.0 in group B (Figure 2) The overall mean BMI was 27, group A median was 26.5 and group B 27.

**Table 1:**
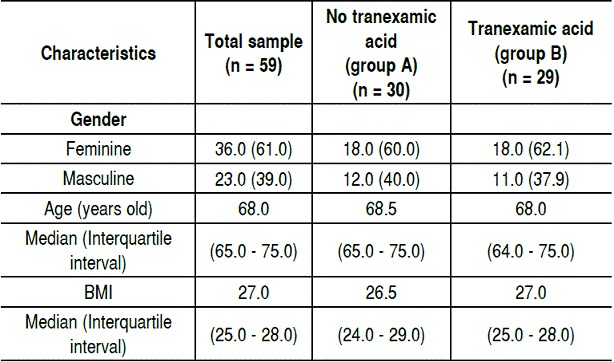
Gender, age and BMI.

All data is presented as n (%), unless otherwise stated.

**Figure 3:**
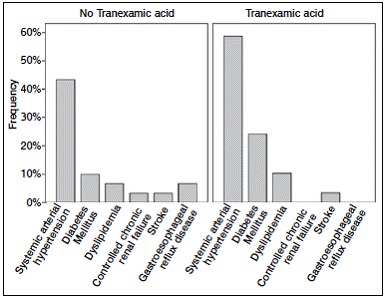
Comorbidities per group.

Table 2 shows the patients' clinical data. Of the patients, 30 had impaired right knee, while 29 had impaired left knee. As for comorbidities, systemic arterial hypertension (SAH) was present in 30 (50.5%) patients, 13 in group A (43.3%) and 17 in group B (58.6%). Diabetes Mellitus was present in 10 subjects (16.9%), 3 (10%) in group A and 7 (24.1%) in group B; dyslipidemia was present in 5 patients (8.5%), 2 (6.7%) in group A and 3 (10.3%) in group B. (Figure 3)

**Table 2:**
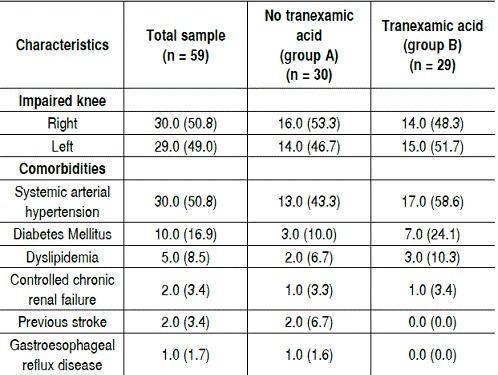
Clinical data.

All data is presented as n (%), unless otherwise stated.

Table 3 shows the clinical data related to surgery. According to the Visual Analogue Scale (VAS)^12^ for pain a lower score was obtained for group B (3.7 - 3.9) both 24h and 48h, respectively, when compared to group A, which had higher values ​​on pain scale, 5.3 at 24h and 4.8 in 48h. The load with support was assessed by patient analysis in standing position, with the aid of a walker, an exercise assisted by the physiotherapist. The average values at 0 and 72h were, respectively, 10.3% and 13.8% for group A and 2.7% and 13.3% for group B. The evaluation by the WOMAC^11^ scale showed no statistically significant difference between the two groups postoperatively. Complications were more frequent among patients in group A: nausea (20%), vomiting (13.3%) and headache (13.3%). However, incontinence (6.9%) was higher among patients in group B. The range of motion showed lower decline among patients in group B, 0°-120° preoperatively to 0°-110° postoperatively. Conversely, patients in group A presented lower range of motion, 0°-120° preoperatively and 0°-100° postoperatively.

**Table 3:**
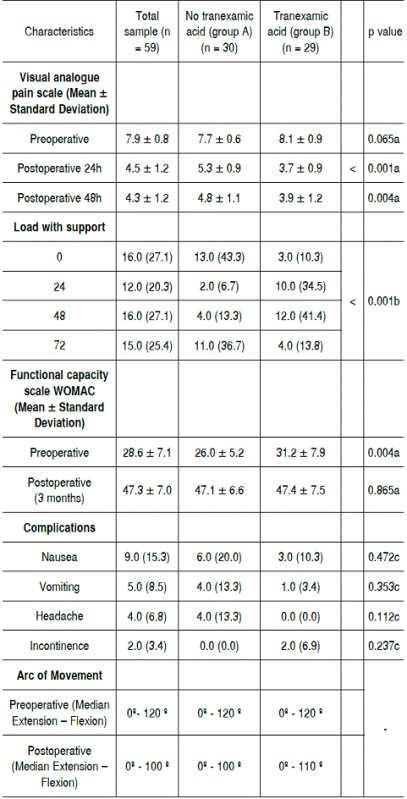
Clinical data related to surgery.

a: Student t-test; b:Chi-square test; c: Fisher's exact test

**Figure 4:**
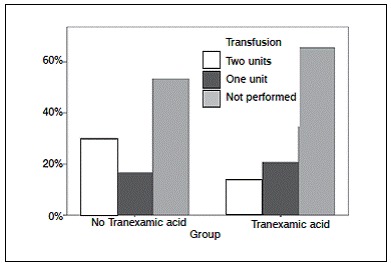
Transfusion per group.

**Figure 5:**
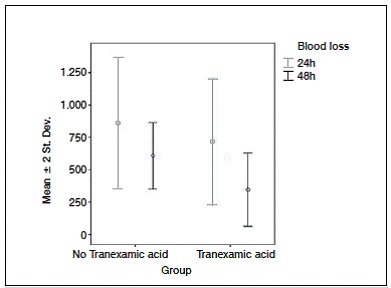
Blood loss per group.

Table 4 shows the hemodynamic parameters, including hemoglobin, quantification of blood transfusions and blood loss. Of the 59 patients undergoing TKA, 35 (59.3%) did not require blood transfusion. Of these, 65.5% were in group B, which were treated with TA. Among patients who required blood transfusion, 18.6% used one blood unit, 16.7% in group A and 20.7% in group B. For those who required two units of blood transfusion, 30% were in group A and 13.8% in group B. (Figure 4) Regarding the amount of blood after TKA in the first 24h, we obtained average of 791.5 ml in general, but group B lost on average 719 ml. For bleeding 48h after TKA, the average blood loss was 479.8 ml in general, 608.3 ml in group A and 346.9 ml in group B. Therefore, patients who used TA showed statistically significant less bleeding, nearly half than those who did not. (Figure 5) 

**Table 4:**
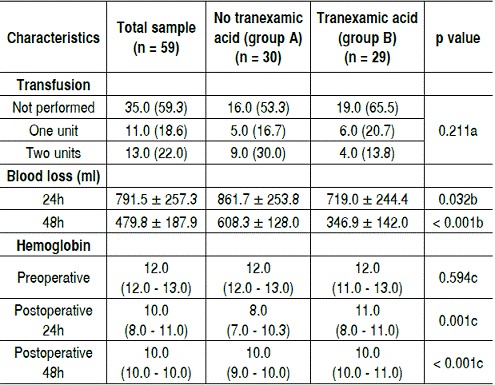
Hemodynamic and hemoglobin parameters.

a: Chi-square test; b: Student t-test; c: Mann Whitney test

**Figure 6:**
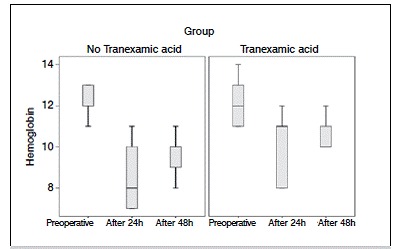
Hemoglobin per time and group.

The rate of bleeding in both groups was similar to the rates reported in the literature, ranging from 900 to 2000 ml. The median hemoglobin rate preoperatively was 12g/dl in both groups. In the first 24h after surgery there was a decreased hemoglobin value in group B to 11 g/dl, while group A has dropped to 8 g/dl, a statistically significant difference. (Figures 6 and ^7^)

**Figure 7:**
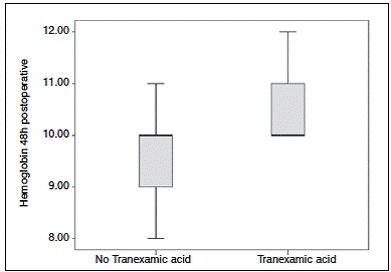
Boxplot: Hemoglobin 48h postoperative.

## DISCUSSION

Regarding the amount of bleeding in the first 24h in TKA procedure, patients who used TA obtained a bleeding rate with values ​​close to half of the group had not use TA, a statistically significant difference, confirmed by the current literature, which shows a blood loss of 900ml and 2000ml.^13^ Tan et al. ^9^ demonstrated in their meta-analysis review bleeding rates with TA of 373.8 ± 264.6 ml which is close to values found in this study, 346.9 ± 142.0 ml.

In 2013, Kim et al.^7^ conducted a study in order to examine the effectiveness of TA in reducing blood loss and transfusion rate in unilateral and bilateral TKA. One hundred and eighty patients undergoing unilateral arthroplasty and 146 patients undergoing bilateral procedure were included. This study showed that TA decreased total blood loss, but the effects on the rate of blood transfusion may differ, according to the type arthroplasty. In patients undergoing bilateral arthroplasty there was a decrease in the rate of blood transfusion, which was not observed in patients undergoing unilateral arthroplasty.

Kakar et al.^1^ and Kim et al.^7^ demonstrated similar results with 24 patients undergoing unilateral TKA and 26 patients operated bilaterally. That study also concluded that TA reduced blood transfusions post-operatively in both groups, which diverges from the conclusion exposed by Kim et al.,^7^ which reports reduction of blood transfusion rates in patients undergoing bilateral procedure. Other parameters regarding use of TA to reduce bleeding include the dose used and the application method.

Roy et al.^14^ studied 50 patients undergoing unilateral TKA, evaluating the efficacy of intraarticular topic TA in reducing blood loss when compared to the control group. They also observed reduction in blood transfusion in patients treated with TA undergoing TKA. The parameters used for this analysis were decrease of hemoglobin levels. The results showed that the control group received six times more blood transfusions than the TA group.

Aguilera et al.^15^ analyzed 172 patients undergoing primary TKA. Their study was based on analysis of the effectiveness of IV TA and fibrin glue compared to hemostasis using Tissucol^(r)^ (fibrinogen and thrombin).^15^ The results of this study showed a lower blood loss in the group using TA when compared to the group using Tissucol^(r)^. Comparing fibrin glue associated with TA, there were not benefits with the association. No complications such as deep vein thrombosis/lung thromboembolism, myocardial infarction or stroke were reported. In this study there was a small number of patients in each group that may have influenced the findings.

Pachauri et al.^16^ conducted a randomized clinical trial with 99 patients to evaluate the efficacy of TA in postoperative TKA, demonstrating a significant reduction in hemoglobin decrease in these patients.The use of IV TA presents satisfactory results, shown consistently on the literature. The IV medication has the advantage to be more quickly as compared to the topical application method. In some countries, such as Germany for example, use of topical TA is not allowed, as indicated in the medication leaflet. Similarly, it is not clear in the literature which is the effect of the medication in contact with bone or muscle tissue. Despite studies showing similar bleeding rates in both methods^17^, the knowledge on IV administration mechanism is more consistent. There are four TA administration methods: intramuscular, oral, intravenous and topic, the last two being the most widely used in Brazil. The time taken by TA to reach maximum plasma level is 30 min for intramuscular injection, 2h for oral administration and 5-15 min intravenously. The IV application was found the best method to obtain a rapid effect of TA and maintaining its therapeutic concentration during and after surgery.^4^

The use of TA did not cause any increase in side effects such as thromboembolic events, therefore suggesting a satisfactory degree of security. The use of IV TA as hemostasis mechanism can reduce costs, decrease the time of hospitalization, and avoid use of autologous blood and its complications, as well as enabling earlier rehabilitation of patients.^2^

In this study, the median hemoglobin level preoperatively showed minimal decrease when using TA. On the other hand, the group that did not use the drug showed a significant decrease in serum hemoglobin levels, requiring blood transfusion. The preservation of hemoglobin levels after a surgery with great bleeding potential gives IV TA a major impact on decreased need for blood transfusion and possible complications.^18^ The literature confirms this hypothesis and demonstrates the importance of routine use of TA in TKA surgeries.^19^

The present study demonstrated that there is a prevalence of females over males, as well as the involvement of the right knee over the left, in accordance with studies by Eubanks et al. ^20^ and Carvalho et al.^8^ Patients in the sample were pre-determined by age of inclusion criteria (60 - 80 years old), which is the most susceptible age group for osteoarthrosis.^5^

Patients who are referred to TKA often have comorbidities and systemic hypertension is the most prevalent, followed by obesity, dyslipidemia, diabetes mellitus, congestive heart failure, cerebrovascular accident and tumors.^9^ This study corroborates these data, showing systemic hypertension as the most prevalent comorbidity in the population, followed by diabetes mellitus, in accordance with the prevalence of chronic diseases in the population aged over 60 years. Other comorbidities such as dyslipidemia, controlled chronic renal failure, previous cerebrovascular accident and gastroesophageal reflux disease were present to a lesser extent, also complying with their frequency in the elderly population.^8^

The study showed a significant reduction in pain through the visual analogue scale^10^ with statistically significant data, especially in the first 24h after surgery, showing the benefit of TA in promoting bleeding control and pain reduction in the postoperative period. These data can be related to decreased hemarthrosis and knee joint volume. The main limitation of this study is the number of patients, which was established based on previous studies with similar samples and on the flow of surgeries at our institution. The comparison between different TA application methods still needs to be researched in order to get a more accurate conclusion regarding the most effective application method. However, recent studies consistently confirmed that TA reduces the amount of bleeding and need for blood transfusions in TKA surgeries.^9^ ^,^ ^13^ ^,^ ^18^

## CONCLUSION

The use of tranexamic acid intravenously in total knee arthroplasty reduces postoperative bleeding rates significantly. The findings of this study demonstrated a reduction in total blood loss and drop of hemoglobin levels, consequently reducing the need for blood transfusions.
